# Antiplatelet effects of aspirin vary with level of P2Y_12_ receptor blockade supplied by either ticagrelor or prasugrel

**DOI:** 10.1111/j.1538-7836.2011.04453.x

**Published:** 2011-10

**Authors:** N S KIRKBY, P D M LEADBEATER, M V CHAN, S NYLANDER, J A MITCHELL, T D WARNER

**Affiliations:** * The William Harvey Research Institute, Barts and the London School of Medicine and Dentistry, Queen Mary University of LondonCharterhouse Square, London; † Cardiothoracic Pharmacology, National Heart and Lung InstituteImperial College, London, UK; ‡ Bioscience Department, AstraZeneca R&DMölndal, Sweden

‘Dual antiplatelet therapy’, comprising aspirin and a P2Y_12_ receptor inhibitor, is firmly established for the secondary prevention of thrombotic events with the rationale that they inhibit thromboxane A_2_- (TxA_2_) and ADP-P2Y_12_-dependent pathways of platelet activation, respectively. We have recently reported that strong P2Y_12_ receptor blockade alone, however, can provide inhibition of platelet aggregation to a broad range of agonists that is not further enhanced by aspirin [1]. While the clinical relevance of these observations is unclear, we have speculated that administration of aspirin to individuals achieving sufficiently strong P2Y_12_ receptor blockade, has the potential to produce effects secondary to inhibition of cyclo-oxygenase at non-platelet sites, without providing additional antithrombotic activity [2]. The degree of P2Y_12_ pathway blockade that is achieved in clinical practice, however, is quite variable [3], reflecting both the choice of drug and large inter-individual differences in drug metabolism [4]. Here, we have extended our previous observations of the interactions between aspirin and strong P2Y_12_ blockade [1] by considering what additional anti-aggregatory effects aspirin provides when only partial P2Y_12_ blockade is achieved, which may better reflect the clinical reality of these drugs.

We measured aggregation responses of platelet-rich plasma (PRP), using 96-well plate light transmission aggregometry, as previously described [1]. Blood was collected by venepuncture into tri-sodium citrate (0.32% final) from healthy volunteers who had abstained from non-steroid anti-inflammatory drug consumption for 14 days. To model the effects of P2Y_12_ blockade and cyclo-oxygenase inhibition *in vitro*, PRP was incubated with the irreversible thienopyridine P2Y_12_ inhibitor, prasgurel-active metabolite (PAM; 0.1–10 μmol L^−1^), the reversible, cyclo-pentyl-triazolo-pyrimidine P2Y_12_ antagonist, ticagrelor (0.1–10 μmol L^−1^) and/or aspirin (1–100 μmol L^−1^) for 30 min at 37 °C before addition of the agonist. Additional methodological details are provided as online supplementary information.

Using this approach we determined the inhibitory potencies of ticagrelor, PAM and aspirin against aggregations induced by ADP (0.625–20 μmol L^−1^), the thromboxane-mimetic U46619 (0.1–30 μmol L^−1^) and arachidonic acid (0.1–1 mmol L^−1^). Both ticagrelor and PAM caused concentration-dependent inhibition of aggregations induced by ADP (Fig. S1), with ticagrelor displaying greater potency than PAM (log IC_50_ values for inhibition of aggregation to 20 μmol L^−1^ ADP: ticagrelor, −6.46; PAM, −5.64). Notably, the potency of ticagrelor, but not PAM, varied with the concentration of ADP (e.g. log IC_50_ values for inhibition of aggregation to 2.5 μmol L^−1^ ADP: ticagrelor, −7.05; PAM, −5.63). Aspirin, at concentrations up to 100 μmol L^−1^, was without significant effect upon ADP-induced aggregations. Ticagrelor and PAM, but not aspirin, produced complete, concentration-dependent inhibition of platelet aggregations induced by U46619 (Fig. S2) with similar potency as for inhibition of ADP-induced aggregations (log IC_50_ values for inhibition of aggregation to 30 μmol L^−1^ U46619; ticagrelor, −6.24; PAM, −5.25). This is consistent with earlier reports that the second, irreversible wave of platelet aggregation that follows TP receptor activation is dependent upon platelet-derived ADP acting upon platelet P2Y_12_ receptors [5]. Ticagrelor and PAM, as well as aspirin, also produced complete, concentration-dependent inhibitions of platelet aggregations induced by AA (Fig. S3; log IC_50_ values for inhibition of aggregation to 1 mmol L^−1^ AA: ticagrelor, −6.88; PAM, −6.00; aspirin, −5.20). When the production of TxA_2_ accompanying platelet aggregation induced by AA was measured (by immunoassay for the levels of TxB_2_), ticagrelor, PAM and aspirin were all found to cause concentration-dependent reductions in TxA_2_ formation (Fig. S3; log IC_50_ values for inhibition of aggregation to 1 mmol L^−1^ AA: ticagrelor, −6.88; PAM, −5.985; aspirin, −5.51). This is in agreement with our early findings [1, 6], and indicates that P2Y_12_ receptors are important in supporting both the activation mechanisms of platelets that drive TxA_2_ formation and pathways downstream of the TP receptor.

To explore further the interactions between P2Y_12_ receptors and the TxA_2_ system in platelets, we examined the effect of aspirin on aggregation induced by a range of agonists in the presence of concentrations of ticagrelor or PAM producing different degrees of partial P2Y_12_ blockade. From the inhibitor curves to ADP described above, concentrations of ticagrelor showing approximate IC_5_ (0.03 μmol L^−1^), IC_10_ (0.1 μmol L^−1^), IC_50_ (0.3 μmol L^−1^) and IC_90_ effects (3 μmol L^−1^) were combined with 30 μmol L^−1^ aspirin, a concentration approximately equivalent to the peak plasma levels following ingestion of a 75–100 mg dose of aspirin. In these experiments, responses to AA (Fig. 1A) were found to be completely inhibited by ticagrelor at the higher two concentrations (representing ∼IC_50_ and IC_90_ for ADP-induced aggregation) without the need for aspirin. The lower two concentrations of ticagrelor (representing ∼IC_5_ and IC_10_ for ADP-induced aggregation) also produced substantial, but incomplete, inhibitions (Table S1). Ticagrelor also inhibited aggregations induced by ADP, collagen, epinephrine, the PAR-1 activating peptide, TRAP-6 (SFLLRN-amide) and U46619, in a concentration-dependent manner (Fig. 1).

**Figure 1:**
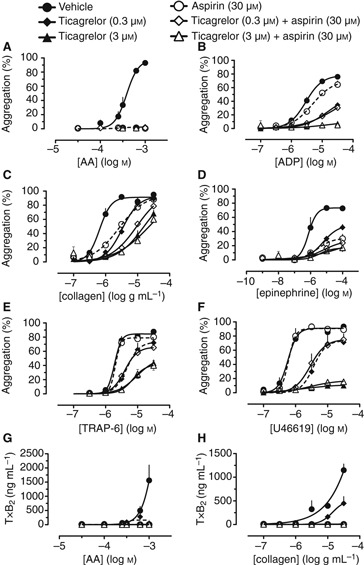
Concentration-response curves for the inhibition by combinations of ticagrelor (0.3 or 3 μmol L^−1^) and aspirin (30 μmol L^−1^) of platelet aggregations induced by (A) arachidonic acid (AA), (B) ADP, (C) collagen, (D) epinephrine, (E) TRAP-6 or (F) U46619, and of platelet TxB_2_ formation induced by (G) AA and (H) collagen. *n* = 4.

When applied alone, aspirin inhibited aggregations induced by AA (Fig. 1A), collagen (Fig. 1C) and epinephrine (Fig. 1D), and showed a weak effect against ADP (Fig. 1B) but did not alter aggregations induced by TRAP-6 (Fig. 1E) or U46619 (Fig. 1F). In contrast, aspirin did augment the anti-aggregatory effects of the lower three concentrations of ticagrelor (achieving incomplete P2Y_12_ inhibition) against both collagen (Fig. 1C) and epinephrine (Fig. 1D). In the presence of the highest tested concentration of ticagrelor (3 μmol L^−1^; ∼IC_90_ for ADP-induced aggregation), aspirin provided no additional anti-aggregatory effects to those of ticagrelor against aggregations to any agonist (Fig. 1). In agreement with earlier experiments, production of TxA_2_ induced by either AA (Fig. 1G) or collagen (Fig. 1H) was partially inhibited by 0.3 μmol L^−1^ ticagrelor (∼IC_50_ for ADP-induced aggregation) and abolished by 3 μmol L^−1^ ticagrelor (∼IC_90_ for ADP-induced aggregation). We have previously reported that TxA_2_ production in response to epinephrine is inhibited by P2Y_12_ blockade in the same manner for AA and collagen [1]. Aspirin (30 μmol L^−1^) either alone or in combination with ticagrelor also completely inhibited TxA_2_ production to either agonist (Fig. 1G,H). A similar pattern of results was obtained using equivalent inhibitory concentrations of PAM in place of ticagrelor (Table S2), and when the concentration of aspirin was increased to 120 μmol L^−1^ (Tables S1 and S2).

These studies show that ticagrelor and PAM inhibit platelet aggregation induced by a range of platelet agonists through a mechanism consistent with blockade of platelet P2Y_12_ receptors and that ticagrelor is more potent than PAM in this regard. As well as inhibiting aggregation following from direct activation of P2Y_12_ receptors by the addition of exogenous ADP, ticagrelor and PAM inhibited aggregations resulting from stimulation of platelets with AA, a response which is well characterized as being TxA_2_ dependent. In addition to inhibiting platelet responses to endogenously produced TxA_2_, ticagrelor and PAM also inhibited the production of TxA_2_ by platelets [6]. Interestingly, ADP itself is a poor stimulus for TxA_2_ production [1], suggesting that released ADP, acting on the P2Y_12_ receptor, acts to potentiate the stimulation of TxA_2_ synthesis by other signaling pathways activated in parallel. These results are consistent with the idea that whereas aspirin may inhibit just the TxA_2_-dependent pathway of platelet activation, ticagrelor and PAM can inhibit both the ADP-P2Y_12_-dependent and the TxA_2_-dependent pathways of platelet aggregation. The observation that aspirin adds anti-aggregatory effects to partial, but not complete, P2Y_12_ receptor blockade, further supports this idea.

Taken together these results demonstrate that rather than ADP-P2Y_12_ and TxA_2_ pathways acting independently, the TxA_2_-dependent pathway is dependent upon the ADP-P2Y_12_ pathway both for the production of TxA_2_ and fundamentally for the irreversible aggregation that follows activation of TP receptors. If these data accurately model the situation *in vivo*, this may have important implications for the use of dual antiplatelet therapy using potent P2Y_12_ antagonists in clinical practice [2]. For example, one could postulate that addition of aspirin could produce side-effects secondary to inhibition of cyclo-oxygenase at non-platelet sites, as has recently become apparent for non-steroid anti-inflammatory drugs, while providing little additional anti-aggregatory effect [7, 8]. Clearly, the validity of this hypothesis remains to be determined by clinical studies.

## Supporting Information

**Figure S1.** Concentration-response curves forthe inhibition by (A) ticagrelor(0.1–10 μmol L^−1^), (B)prasugrel active metabolite (PAM;0.1–10 μmol L^−1^) and (C)aspirin (1–100 μmol L^−1^) ofplatelet aggregation induced by ADP(0.625–20 μmol L^−1^).*n* = 4.

**Figure S2.** Concentration-response curves forthe inhibition by (A) ticagrelor(0.1–10 μmol L^−1^), (B)prasugrel active metabolite (PAM;0.1–10 μmol L^−1^) and (C)aspirin (1–100 μmol L^−1^) ofplatelet aggregation induced by U46619(0.1–30 μmol L^−1^).*n* = 4.

**Figure S3.** Concentration-response curves forthe inhibition by (A, B) ticagrelor(0.1–10 μmol L^−1^), (C, D)prasugrel active metabolite (PAM;0.1–10 μmol L^−1^) and (E, F)aspirin (1–100 μmol L^−1^) ofplatelet aggregation (A, C, E) and TxA_2_ release (B, D,F; measured as TxB_2_) induced by arachidonic acid (AA;0.03–1 mmol L^−1^).*n* = 4.

**Table S1.** Area under the concentration-responsecurve summary data for the effect of combinations of ticagrelor(0.03, 0.1, 0.3 and 3 μmol L^−1^) andaspirin (30 and 120 μmol L^−1^) onplatelet aggregation (A–C) and platelet TxA_2_release (D; measured as TxB_2_).

**Table S2.** Area under the concentration-responsecurve summary data for the effect of combinations ofprasugrel-active metabolite (PAM; 0.5, 1, 2 and10 μmol L^−1^) and aspirin (30 and120 μmol L^−1^) on platelet aggregation(A–C) and platelet TxA_2_ release (D; measured asTxB_2_).
