# Supplementary material for: Antiplatelet effects of aspirin vary with level of P2Y12 receptor blockade supplied by either ticagrelor or prasugrel
Source: J Thromb Haemost. 2011 Oct;9(10):2103–5. doi: 10.1111/j.1538-7836.2011.04453.x (PMC3399085; doi:10.1111/j.1538-7836.2011.04453.x)
Supplement: Supplementary file 1 [file jth0009-2103-SD1.doc]

ANTI-PLATELET EFFECTS OF ASPIRIN VARY WITH LEVEL OF P2Y_12_ RECEPTOR BLOCKADE SUPPLIED BY EITHER TICAGRELOR OR PRASUGREL

Nicholas S. Kirkby^†^*, Philip D.M. Leadbeater^†^*, Melissa V. Chan*, Sven Nylander**, Jane A. Mitchell^†^ and Timothy D. Warner*

From *The William Harvey Research Institute, Barts & the London School of Medicine & Dentistry, Queen Mary University of London, Charterhouse Square, London, EC1M 6BQ, U.K.; ^†^Cardiothoracic Pharmacology, National Heart and Lung Institute, Imperial College, London, SW3 6LY, U.K.; and **Bioscience Department, AstraZeneca R&D Mölndal, Sweden.

**Supplementary Information**

**Supplementary Methods**

*Blood Collection*

Blood was collected from healthy volunteers (St Thomas’s Hospital Research Ethics Committee, reference 07 / Q0702 / 24), who had abstained from NSAID consumption for the preceding 14 days, by venepuncture into tri-sodium citrate (3.2%, 1:9 v/v; Sigma, Poole, Dorset, U.K.). Platelet-rich plasma (PRP) was obtained by centrifugation at 175 x g for 15 minutes at 25°C. Platelet-poor plasma (PPP) was obtained by centrifugation of PRP at 15000 x g for 2 minutes. Platelet counts were made to confirm normal platelet number, 2-4x10^8^ cells mL^-1^, but samples were not adjusted. All experiments were completed within 2h of blood collection.

*96-Well Plate Light Transmission Aggregometry*

To assess the aggregation of platelets in 96-well plates a modified light transmission method was used [1, 16-18]. Briefly, 100µL samples of PRP were pre-incubated for 30 minutes at 37°C with ticagrelor or PAM (0.1-10µmol L^‑1^), and/or aspirin (30-120µmol L^‑1^), or vehicle (0.5% DMSO) before being placed into the individual wells of a 96-well microtitre plate (Nunc, Lutterworth, Leicestershire, U.K.) containing 10µL of vehicle or agonist: adenosine diphosphate (ADP; 0.1-30µmol L^-1^; LabMedics, Salford, Manchester, U.K.), arachidonic acid (AA; 0.03-1mmol L^-1^; Sigma, Poole, U.K.), Horm collagen (0.1-30µg mL^-1^; Nycomed, Linz, Austria), epinephrine (0.001-100 µmol L^-1^; LabMedics), the PAR-1 activating peptide TRAP‑6 amide (SFLLRN-amide; 0.1-30µmol L^-1^; Bachem, Bubendorf, Switzerland), and the stable TxA_2_-mimetic U46619 (0.1‑30µmol L^-1^; Cayman Chemical Company, Ann Arbor, MI, U.S.A.). These agonists were chosen as together they represent the range of platelet agonists most widely used in *in vitro* platelet testing [19]. The plate was then placed into a 96-well plate reader (Tecan Sunrise) at 37°C, and absorbance was measured at 595nm every 15 seconds for 16 minutes with vigorous shaking between readings. Percentage aggregation was calculated with reference to the absorbance of PPP as a surrogate for 100% aggregation. Graphs shown are for aggregation responses at 16 minutes, as we have described previously [1, 16-18].

*Thromboxane B_2_ ELISA*

At the end of the PRP aggregation monitoring, cyclo-oxygenase activity was halted by the addition of 1mmol L^-1^ diclofenac (Sigma), the samples were centrifuged at 1300g for 10 minutes at 5°C, and the supernatants removed and frozen. TxB_2_ levels in the supernatant, as a measure of TxA_2_ formation, were determined using selective ELISA (Cayman chemical, USA).

*Statistical Analysis*

All statistical analyses were conducted using GraphPad Prism v5 (GraphPad Software Inc, CA, USA). Agonist concentration response curves were plotted and analysed according to the four parameter logistic equation:

Y=Bottom+(Top-Bottom)/1+10^(logEC_50_-X)*HillSlope)

Data were analysed as area under the concentration-response curve and statistical significance determined by one-way ANOVA with Bonferroni post-tests. All data is expressed as mean ± standard error of the mean (SEM); n values for each data point refer to the number of different donors that supplied platelets. Differences were considered significant if p<0.05.

**Supplementary Figure 1**

Concentration-response curves for the inhibition by A) ticagrelor (0.1-10μmol L^-1^), B) prasugrel active metabolite (PAM; 0.1-10μmol L^-1^), and C) aspirin (1-100μmol L^-1^) of platelet aggregation induced by ADP (0.625-20μmol L^-1^). n=4.

**Supplementary Figure 2**

Concentration-response curves for the inhibition by A) ticagrelor (0.1-10μmol L^-1^), B) prasugrel active metabolite (PAM; 0.1-10μmol L^-1^), and C) aspirin (1-100μmol L^-1^) of platelet aggregation induced by U46619 (0.1-30μmol L^-1^). n=4.

**Supplementary Figure 3**

Concentration-response curves for the inhibition by A, B) ticagrelor (0.1-10μmol L^-1^), C, D) prasugrel active metabolite (PAM; 0.1-10μmol L^-1^), and E, F) aspirin (1-100μmol L^-1^) of platelet aggregation (A, C, E) and TxA_2_ release (B, D, F; measured as TxB_2_) induced by arachidonic acid (AA; 0.03-1mmol L^-1^). n=4.

| **A** | **[ticagrelor]** | **vehicle** | **vehicle** | **0.03µmol L^-1^** | **0.03µmol L^-1^** | **0.1µmol L^-1^** | **0.1µmol L^-1^** |
| --- | --- | --- | --- | --- | --- | --- | --- |
|  | **[Aspirin]** | **vehicle** | **30µmol L^-1^** | **vehicle** | **30µmol L^-1^** | **vehicle** | **30µmol L^-1^** |
|  | AA | 70.2±14.2 | 4.2±1.2^*^ | 42.8±12.8 | 2.6±0.7^*^ | 28.7±13.2^*^ | 6.3±1.8^*^ |
|  | ADP | 151.8±22.0 | 120.8±18.1 | 141.2±20.4 | 118.3±18.0 | 107.0±12.1 | 85.7±11.1 |
|  | Collagen | 148.6±15.6 | 102.8±11.2 | 143.3±10.6 | 102.6±12.4 | 124.7±9.6 | 82.9±6.8^*^ |
|  | Epinephrine | 220.1±26.4 | 90.7±12.7^*^ | 196.6±16.2 | 71.5±14.6^*†^ | 143.9±19.8^*^ | 66.9±10.2^*†^ |
|  | TRAP-6 | 111.3±8.7 | 107.5±4.28 | 113.4±5.6 | 109.4±4.3 | 112.3±6.3 | 86.3±10.2 |
|  | U46619 | 168.6±14.3 | 164.2±6.34 | 168.2±9.4 | 162.0±7.1 | 146.6±13.1 | 140.3±11.8 |
|  |  |  |  |  |  |  |  |
| **B** | **[ticagrelor]** | **vehicle** | **vehicle** | **0.3µmol L^-1^** | **0.3µmol L^-1^** | **3µmol L^-1^** | **3µmol L^-1^** |
|  | **[Aspirin]** | **vehicle** | **30µmol L^-1^** | **vehicle** | **30µmol L^-1^** | **vehicle** | **30µmol L^-1^** |
|  | AA | 44.7±5.2 | 3.5±0.40^*^ | 2.1±0.55^*^ | 3.0±0.8^*^ | 2.1±0.8^*^ | 2.6±1.0^*^ |
|  | ADP | 81.4±4.1 | 61.4±2.5^*^ | 24.1±2.7^*^ | 23.5±3.0^*^ | 6.7±0.8^*^ | 9.8±1.1^*^ |
|  | Collagen | 154.4±7.6 | 105.1±4.8^*^ | 95.4±5.2^*^ | 65.7±1.4^*†^ | 49.6±6.7^*^ | 48.5±2.3^*^ |
|  | Epinephrine | 159.3±13.0 | 52.8±9.4^*^ | 66.7±7.8^*^ | 40.9±6.4^*^ | 28.3±6.7^*^ | 33.0±4.1^*^ |
|  | TRAP-6 | 104.5±3.74 | 97.0±2.0 | 61.8±6.7^*^ | 60.1±7.8^*^ | 30.0±3.5^*^ | 28.7±6.0^*^ |
|  | U46619 | 158.7±10.6 | 157.3±6.5 | 73.3±12.6^*^ | 79.8±16.1^*^ | 14.2±2.43^*^ | 19.5±1.3^*^ |
|  |  |  |  |  |  |  |  |
| **C** | **[ticagrelor]** | **vehicle** | **vehicle** | **0.3µmol L^-1^** | **0.3µmol L^-1^** | **3µmol L^-1^** | **3µmol L^-1^** |
|  | **[Aspirin]** | **vehicle** | **120µmol L^-1^** | **vehicle** | **120µmol L^-1^** | **vehicle** | **120µmol L^-1^** |
|  | AA | 79.8±4.4 | 3.5±1.1^*^ | 3.3±0.3^*^ | 2.0±0.4^*^ | 3.3±0.9^*^ | 3.4±0.9^*^ |
|  | ADP | 96.2±7.6 | 75.8±6.1^*^ | 24.3±1.8^*^ | 21.8±1.0^*^ | 8.2±0.5^*^ | 9.4±1.2^*^ |
|  | Collagen | 176.1±6.1 | 121.2±4.9^*^ | 117.2±2.0^*^ | 76.6±3.0^*†^ | 76.7±2.6^*^ | 54.1±0.7^*†^ |
|  | Epinephrine | 207.6±24.7 | 61.0±7.6^*^ | 84.6±8.6^*^ | 38.3±4.7^*^ | 48.8±2.4^*^ | 35.3±4.1^*^ |
|  | TRAP-6 | 121.0±4.9 | 113.1±3.4 | 77.7±4.9^*^ | 70.2±4.4^*^ | 41.3±3.3^*^ | 38.3±2.7^*^ |
|  | U46619 | 177.4±2.9 | 175.1±8.2 | 104.8±8.1^*^ | 111.3±4.4^*^ | 21.7±2.5^*^ | 24.1±1.4^*^ |
|  |  |  |  |  |  |  |  |
| **D** | **[ticagrelor]** | **vehicle** | **vehicle** | **0.3µmol L^-1^** | **0.3µmol L^-1^** | **3µmol L^-1^** | **3µmol L^-1^** |
|  | **[Aspirin]** | **vehicle** | **30µmol L^-1^** | **vehicle** | **30µmol L^-1^** | **vehicle** | **30µmol L^-1^** |
|  | AA | 320.5±97.8 | 6.5±2.00^*^ | 106.7±40.0^*^ | 3.1±0.7^*^ | 8.9±3.9^*^ | 3.2±0.7^*^ |
|  | Collagen | 657.5±143.7 | 8.5±1.1^*^ | 207.8±56.8^*^ | 6.8±2.5^*^ | 11.7±4.4^*^ | 4.6±1.1^*^ |

**Supplementary Table 1**

Area under the concentration-response curve summary data for the effect of combinations of ticagrelor (0.03, 0.1, 0.3 and 3 µmol L^-1^) and aspirin (30 and 120 µmol L^-1^) on platelet aggregation (A-C) and platelet TxA_2_ release (D; measured as TxB_2_). In A-C units are log(mol.L^-1^).% for AA, ADP, epinephrine, TRAP-6 and U46619, and log(grams.mL^-1^).% for collagen. In D units are log(mol.L^-1^).ng.mL^-1^ for AA and log(mol.L^-1^).ng.mL^-1^ for collagen. Data is presented as mean ± standard error of the mean. *, p<0.05 compared to vehicle; ^†^, p<0.05 compared to the response with the same concentration of ticagrelor in the absence of aspirin; by one-way ANOVA.

| **A** | **[PAM]** | **vehicle** | **vehicle** | **0.5µmol L^-1^** | **0.5µmol L^-1^** | **1µmol L^-1^** | **1µmol L^-1^** |
| --- | --- | --- | --- | --- | --- | --- | --- |
|  | **[Aspirin]** | **vehicle** | **30µmol L^-1^** | **vehicle** | **30µmol L^-1^** | **vehicle** | **30µmol L^-1^** |
|  | AA | 38.2±10.2 | 1.8±0.6^*^ | 17.7±9.0 | 2.8±0.5^*^ | 6.9±3.9^*^ | 3.6±0.9^*^ |
|  | ADP | 136.8±6.1 | 112.9±2.6 | 111.4±6.1 | 93.8±5.1^*^ | 82.9±8.8^*^ | 72.0±6.1^*^ |
|  | Collagen | 150.8±11.1 | 101.7±4.5^*^ | 141.3±8.2 | 96.3±4.1^*†^ | 124.7±7.5 | 81.2±4.5^*†^ |
|  | Epinephrine | 182.3±34.2 | 61.3±23.7 | 128.1±41.7 | 45.9±18.8^*^ | 86.2±34.0 | 39.2±16.8^*^ |
|  | TRAP-6 | 111.3±8.7 | 107.5±4.2 | 105.3±7.4 | 98.3±9.8 | 95.8±10.9 | 90.7±9.4 |
|  | U46619 | 157.9±9.9 | 144.4±12.4 | 135.3±15.6 | 135.0±14.8 | 118.7±19.3 | 112.3±18.2 |
|  |  |  |  |  |  |  |  |
| **B** | **[PAM]** | **vehicle** | **vehicle** | **2µmol L^-1^** | **2µmol L^-1^** | **10µmol L^-1^** | **10µmol L^-1^** |
|  | **[Aspirin]** | **vehicle** | **30µmol L^-1^** | **vehicle** | **30µmol L^-1^** | **vehicle** | **30µmol L^-1^** |
|  | AA | 39.0±9.6 | 2.1±0.2^*^ | 4.5±1.5^*^ | 3.3±1.6^*^ | 2.0±0.6^*^ | 1.8±0.9^*^ |
|  | ADP | 84.5±9.0 | 69.1±2.9 | 23.6±3.1^*^ | 21.6±3.5^*^ | 6.5±3.0^*^ | 7.8±2.4^*^ |
|  | Collagen | 164.4±13.7 | 117.6±3.8^*^ | 116.4±4.8^*^ | 78.6±3.8^*†^ | 56.0±3.9^*^ | 49.2±2.6^*^ |
|  | Epinephrine | 139.5±24.1 | 45.9±4.0^*^ | 79.0±8.3^*^ | 42.1±6.0^*^ | 29.3±2.8^*^ | 28.6±3.8^*^ |
|  | TRAP-6 | 97.3±8.9 | 89.9±8.6 | 62.5±5.1^*^ | 58.2±3.0^*^ | 17.9±5.0^*^ | 22.7±3.8^*^ |
|  | U46619 | 148.4±5.8 | 160.0±8.6 | 71.0±21.6^*^ | 81.5±22.4^*^ | 12.9±1.2^*^ | 16.6±1.0^*^ |
|  |  |  |  |  |  |  |  |
| **C** | **[PAM]** | **vehicle** | **vehicle** | **2µmol L^-1^** | **2µmol L^-1^** | **10µmol L^-1^** | **10µmol L^-1^** |
|  | **[Aspirin]** | **vehicle** | **120µmol L^-1^** | **vehicle** | **120µmol L^-1^** | **vehicle** | **120µmol L^-1^** |
|  | AA | 34.8±13.6 | 2.4±1.4^*^ | 3.1±0.8^*^ | 2.6±1.0^*^ | 2.2±0.8^*^ | 2.0±1.2^*^ |
|  | ADP | 76.0±6.1 | 53.2±5.4 | 19.3±10.2^*^ | 21.6±7.8^*^ | 6.9±1.8^*^ | 9.5±2.3^*^ |
|  | Collagen | 148.1±1.9 | 99.2±2.6^*^ | 87.9±17.3^*^ | 62.1±7.9^*^ | 61.2±5.4^*^ | 50.9±7.9^*^ |
|  | Epinephrine | 150.7±22.3 | 28.5±7.5^*^ | 45.0±29.5^*^ | 22.8±5.9^*^ | 24.9±7.5^*^ | 21.2±5.5^*^ |
|  | TRAP-6 | 109.8±1.0 | 97.5±4.6 | 67.8±19.0 | 60.4±16.3 | 37.0±6.5^*^ | 39.4±10.4^*^ |
|  | U46619 | 157.1±5.1 | 157.7±4.1 | 72.0±31.2 | 75.8±31.5 | 17.5±3.3^*^ | 27.6±12.7^*^ |
|  |  |  |  |  |  |  |  |
| **D** | **[PAM]** | **vehicle** | **vehicle** | **2µmol L^-1^** | **2µmol L^-1^** | **10µmol L^-1^** | **10µmol L^-1^** |
|  | **[Aspirin]** | **vehicle** | **30µmol L^-1^** | **vehicle** | **30µmol L^-1^** | **vehicle** | **30µmol L^-1^** |
|  | AA | 196.5±28.4 | 4.5±1.9^*^ | 65.5±20.7^*^ | 2.6±0.7^*†^ | 28.3±4.5^*^ | 3.6±1.0^*^ |
|  | Collagen | 341.7±30.9 | 11.5±3.5^*^ | 283.3±33.2 | 15.6±6.9^*†^ | 141.6±31.6^*^ | 11.0±3.3^*†^ |

**Supplementary Table 2**

Area under the concentration-response curve summary data for the effect of combinations of prasugrel-active metabolite (PAM; 0.5, 1, 2 and 10 µmol L^-1^) and aspirin (30 and 120 µmol L^-1^) on platelet aggregation (A-C) and platelet TxA_2_ release (D; measured as TxB_2_). In A-C units are log(mol.L^-1^).% for AA, ADP, epinephrine, TRAP-6 and U46619, and log(grams.mL^-1^).% for collagen. In D units are log(mol.L^-1^).ng.mL^-1^ for AA and log(mol.L^-1^).ng.mL^-1^ for collagen. Data is presented as mean ± standard error of the mean. *, p<0.05 compared to vehicle; ^†^, p<0.05 compared to the response with the same concentration of PAM in the absence of aspirin; by one-way ANOVA.
